# Progress in Nerve Surgery

**Published:** 1903-10-10

**Authors:** 


*(To be continued.)*

### Progress in Nerve Surgery.

**Trophic Changes, the Result of Nerve Injury.**[1]— Dr. Alex. James relates three cases of multiple lipomata, Raynaud's disease and onychia, all the result of trophic disturbance caused by nerve injury. The first case was that of a miner, aged 35, who complained of pain in the back and over the front of the chest with little swellings over the lower part of the trunk. Whilst at work, some two months before admission, bent on his knees, 15 cwt. of coal fell on the top of him. He was knocked flat, was unconscious for some minutes, and shortly afterwards vomited blood and passed blood in the stools. He remained in bed two months and then being very weak in the back came to the infirmary. He complained of pain and stiffness in the spine from the cervical to the lower dorsal region and the spine was tender to pressure. Voluntary motor power was impaired especially in the right arm. The plantar, abdominal and epigastric reflexes were absent, the knee-jerks and ankle-jerks well marked. The gait was slow but unimpaired. There was no girdle pain nor alteration in cutaneous sensibility. The organic reflexes were unimpaired. The peculiar trophic disturbances were found in connection with the skin. They showed themselves as little swellings, varying in size from a pea to a flattened plum, nine in number, situated over the abdomen and back. They were freely movable, fairly firm, and painless on pressure. One of them excised showed the structure of a lipoma on microscopic examination. No alteration took place in the patient's condition during the month he was in the infirmary, but it was vaguely heard afterwards that he became paralysed. The second case was that of a soldier, aged 23, who at the battle of Magersfontein had a gunshot wound of the metacarpo-phalangeal joint of the left middle finger. The wound healed with ankylosis of the joint, but about six weeks later the hand began to feel numb and cold and to show a bluish coloration. Shortly afterwards similar feelings and changes in colour occurred in the right hand. On examination a condition of intense lividity was recognised on both hands extending to a couple of inches above the wrist. Exposure to cold aggravated the condition, whilst heat diminished it somewhat. There was no history of hæmoglobinuria or of any other abnormal condition. Tenderness was elicited by pressure on the parts from the scars upwards, and there was evidently matting in the neighbourhood of the wounds. In the belief that the condition in both hands was the result of irritation of nerves involved in cicatricial tissue the finger was amputated, the scar tissue removed, and the nerves exposed and freed. The wound healed and after a few weeks of treatment with the Greville hot-air apparatus the lividity and other symptoms rapidly disappeared. The last case was that of a young woman, aged 19, who complained of pain and deformity in the nails and finger-tips of the three outer fingers of her left hand. Her father died of phthisis and she herself had suffered from multiple tubercular bone affections necessitating several operations, specially in the neighbourhood of the left elbow. Her present symptoms had started seven or eight years ago, when the fingers mentioned began to feel cold and numb, a black mark appeared at the root of the nail of the index finger, increased in size, and the nail cracked and fell off. The thumb and middle finger soon became affected in the same way and she had to give up her work. On examination the finger nails are greatly thickened, elongated, and curved, and the corresponding finger-tips appeared atrophied. Many different kinds of treatment had been adopted on the assumption that it was tuberculous onychia, but all without the slightest effect. Pain is felt in the affected fingers when she attempts to use them, and this often shoots up to the shoulder. There is extensive cicatrization on the inner side of the elbow joint, and a further cicatrix on the front of the forearm just below the elbow and over the position of the median nerve. Pressure on this causes pain which shoots up and down the nerve. On the assumption that the trophic disturbance in the fingers was due to irritation of the median nerve caused by the scarring, the cicatrix below the elbow was explored and the nerve found fixed in a mass of fibrous tissue. This was freed and the patient cured.

[1] Scot. Med. and Surg. Jour., May, 1903.

